# Acute Immunological Biomarkers for Predicting Chronic Rheumatologic Disease After Chikungunya Virus Infection

**DOI:** 10.3390/tropicalmed10070195

**Published:** 2025-07-11

**Authors:** Anyela Lozano-Parra, Víctor Herrera, Luis Ángel Villar, Silvio Urcuqui-Inchima, Juan Felipe Valdés-López, Elsa Marina Rojas Garrido

**Affiliations:** 1 Grupo Epidemiología Clínica, Escuela de Medicina, Universidad Industrial de Santander UIS, Calle 9 Carrera 27, Bucaramanga 680002, Colombia; vicmaher@uis.edu.co; 2 Centro de Atención y Diagnóstico de Enfermedades Infecciosas (CDI), Fundación INFOVIDA, Cra. 37 No. 51-126, Bucaramanga 680003, Colombia; direccioninvestigacion@cdi.net.co (L.Á.V.); elsa.rojas@cdi.net.co (E.M.R.G.); 3 Grupo Inmunovirología, Departamento de Microbiología y Parasitología, Facultad de Medicina, Universidad de Antioquia UdeA, Calle 70 No. 52-21, Medellín 050010, Colombia; silvio.urcuqui@udea.edu.co (S.U.-I.); felipe.valdes@udea.edu.co (J.F.V.-L.)

**Keywords:** Chikungunya fever, chronic rheumatologic disease, biomarkers, cytokines, chemokines

## Abstract

Early biomarkers are needed to predict the long-term persistence of rheumatical symptoms in patients infected with Chikungunya virus (CHIKV). This nested case-control study aimed to assess immunological factors during the early phases of CHIKV infection to predict the risk of post-CHIK chronic rheumatism (pCHIK-CR) in adult patients of two prospective cohorts. We evaluated 46 febrile patients (median age: 33.5 years; IQR: 19 years; women: 50.0%) with CHIKV infection confirmed during the 2014–2015 outbreak in Santander, Colombia. The participants were classified by a rheumatologist as either cases (pCHIK-CR) or controls (WoRM, without rheumatical manifestations). We quantified serum levels of IL-4, IL-6, IL-8/CXCL-8, IL-27, CCL-2, CXCL-9, CXCL-10, and IgG using Luminex and ELISA assays during the acute and subacute phases of infection. Then, we evaluated the association of these immune factors with the case-control status using piecewise logistic regression adjusted for age and sex. There were non-linear associations between IL-8/CXCL-8, CXCL-9, and CXCL-10 with pCHIK-CR. Increases in the levels of IL-8/CXCL-8 (<35.7 pg/mL), CXCL-9 (≥6000 pg/mL), and CXCL-10 (≥36,800 pg/mL) were significantly associated with a reduced risk of pCHIK-CR (adjusted ORs: 0.85, 0.96, and 0.94, respectively). These results suggest that increases in IL-8/CXCL-8, CXCL-9, and CXCL-10 levels, measured in the early stages of CHIKV infection, may predict a chronic disease risk. This suggests the possibility that an early and strong immune response could contribute to enhancing CHIKV control and potentially reduce the risk of persistent joint symptoms. Given their expression patterns and timing, these three immune factors may be considered promising biomarker candidates for assessing the risk of chronic rheumatologic disease. These findings should be considered as exploratory and validated in additional cohort studies.

## 1. Introduction

Chikungunya fever (CHIKF) is caused by the Chikungunya virus (CHIKV), an RNA virus member of the *Togaviridae* family, *Alphavirus* genus [1,2]. CHIKV genome contains two open reading frames (ORFs). The first ORF encodes a polyprotein that is cleaved by viral proteases into four non-structural proteins—nsP1, nsP2 (protease), nsP3, and nsP4 (RNA-dependent RNA polymerase)—which are involved in viral genome replication and the evasion of the host’s immune response [3]. The second ORF encodes a polyprotein, which is processed by both viral and cellular proteases to produce six structural proteins: capsid (C), envelope (E3, E2, 6K, and E1), and transfer-enhancing protein (TF). These structural proteins are essential for assembling and generating new viral particles [1,2]. CHIKV is classified into three main genotypes: Asian, West African (WA), and East/Central/South African (ECSA) [4].

CHIKV infection progresses through three clinical phases: acute, subacute, and chronic [5]. The acute phase is characterized by an abrupt onset of fever and joint pain, which may be accompanied by myalgia, headache, nausea, vomiting, conjunctivitis, and skin rashes, lasting 5 to 7 days [6,7]. While most patients recover during this phase, some progress to the subacute phase, characterized by persistent joint pain, fatigue, and weakness, lasting from 10 days to 3 months [5]. The chronic phase begins after three months and can persist for years, manifesting as rheumatic symptoms [5,8,9,10].

During the chronic phase, some cases develop post-Chikungunya chronic rheumatism (pCHIK-CR), defined by the persistence of joint and extra-articular symptoms for more than three months after the disease onset or by the emergence of specific immune-mediated inflammatory pathology during follow-up [11,12]. This condition is frequently associated with a reduction in the quality of life and chronic fatigue [13,14,15,16,17,18,19,20,21]. Several patients’ characteristics, such as an age of over 45 years, the female sex, a history of joint pain, and elevated IgG antibody levels have been proposed as prognostic factors for progression to chronicity [22,23]. However, the understanding of the pathogenesis and the identification of prognostic biomarkers for pCHIK-CR remain limited.

After the bite of an infected *Aedes* mosquito, CHIKV infects and replicates in monocytes, macrophages, fibroblasts, and endothelial cells in the skin [7,24,25]. The virus then spreads to lymph nodes and, through the lymphatic and bloodstream circulation, distributes to various tissues, including the synovial tissue [26]. In the joints, it has been suggested that the virus may persist and evade the immune response by establishing immune-privileged niches, contributing to tissue damage [27]. This hypothesis is supported by the detection of viral RNA in synovial macrophages 18 months after infection [27]. At least one study confirmed the presence of the CHIKV glycoprotein E1 in synovial tissue 22–24 months post-infection, even in the absence of viral RNA [28].

Viral persistence leads to the continuous recruitment and activation of monocytes, macrophages, effector T cells, and natural killer (NK) cells, which release pro-inflammatory cytokines (IL-6, IL-8/CXCL-8, CCL-2, and IFNs) that promote fibroblast apoptosis and cartilage destruction [27,29]. Compared to healthy controls, during the acute phase of CHIKV infection, patients show an immune profile characterized by elevated levels of pro-inflammatory cytokines (IFN-α, IFN-γ, IL-2R, IL-6, IL-7, IL-8/CXCL-8, and IL-27), anti-inflammatory cytokines (IL-1Ra and IL-4), chemokines (CCL-2, CXCL-9, and CXCL-10), and growth factors (VEGF, G-CSF, and GM-CSF) in the blood [30]. Given the apparent role of these inflammatory factors in CHIKV progression, further studies are needed to assess their potential as prognostic biomarkers to help stratify the risk of long-term complications. In this context, our study aimed to quantify the serum levels of a set of eight immunological factors to identify potential candidates early in the course of CHIKF (acute and subacute phases) that could serve as immunological biomarkers for predicting progression to pCHIK-CR.

## 2. Materials and Methods

We conducted a nested case-control study within two cohorts to quantify immune factors in the serum of patients during the acute and subacute phases of CHIKV infection to identify immunological biomarkers potentially associated with the development of pCHIK-CR. This section describes each cohort, the eligibility criteria, case and control definitions, the procedure for immunological factor quantification, and the data analysis plan.

### 2.1. Description of Cohorts

Cases of CHIKV infection were identified in two prospective cohorts assembled during the outbreak that occurred between 2014 and 2015 in the municipalities of Capitanejo and Piedecuesta, Colombia, as detailed below:

Capitanejo cohort: Established in 2015 as a part of the response to the CHIKV outbreak in the municipality of Capitanejo [9], this cohort included 105 patients aged 5 to 95 years who sought health care due to persistent joint pain. During the baseline visit (subacute phase), patients underwent a physical examination, and a blood sample was collected to confirm CHIKV infection. In 2017, a subsample of adult patients with confirmed infection was followed and evaluated by a rheumatologist to determine the presence of pCHIK-CR.

Piedecuesta cohort: This study was assembled by merging two cohorts and was conducted in Piedecuesta. One was based on passive surveillance, and the other on active surveillance. The passive surveillance sub-cohort was assembled in 2014 and recruited 839 participants (aged 1 to 55 years) who sought care for acute febrile syndrome at two healthcare institutions [31]. The active surveillance sub-cohort was assembled in 2015 and recruited 2400 healthy participants from the community [32]. This sub-cohort was followed through biweekly phone calls for 3.5 years to identify cases of acute febrile illness. In both sub-cohorts, the participants with febrile illness were clinically assessed within 7 days of the symptoms’ onset (baseline visit) and followed-up 7 to 14 days later to conduct a clinical evaluation and collect a blood sample. In total, the Piedecuesta cohort identified 219 cases of CHIKV infection during the acute phase of the disease. In 2022, the adult participants with a confirmed CHIKV infection were invited to undergo a physical examination that included a musculoskeletal screening using the GALS methodology (gait, upper extremities, lower extremities) conducted by a physician [33,34]. Additionally, a rheumatologist evaluated those patients with an abnormal GALS screening (unrelated to trauma) to determine the presence of pCHIK-CR.

In both the Capitanejo and the Piedecuesta cohorts, CHIKV infection was diagnosed based on a positive result in the NovaLisa^®^ ELISA IgG or IgM tests (NOVATEC Inmunodiagnostica GmbH, Dietzenbach, Germany) or the reverse transcription quantitative polymerase chain reaction (RT-qPCR) test [35]. This diagnostic approach was adopted because the virus was newly introduced to the population during the study period.

### 2.2. Eligibility Criteria

We included adults over 18 years old with confirmed CHIKV infection who attended a follow-up evaluation during the chronic phase of the disease. We excluded participants with a history of rheumatic disease before CHIKV infection, as well as those diagnosed with non-inflammatory pain likely degenerative (NIP-LD) during the follow-up evaluation. We also excluded individuals who did not consent to using their biological samples for future research, as requested in the original cohorts, and those who had no samples collected at baseline for the quantification of immunological factors.

### 2.3. Case-Control Definition

The case-control status was determined based on the clinical evaluation results conducted during the follow-up visit of the cohorts. The median follow-up was 2.2 years for the patients recruited in the Capitanejo cohort and 7.7 years for those recruited in the Piedecuesta cohort. The cases of pCHIK-CR were defined as patients experiencing persistent joint and extra-articular symptoms lasting more than three months after the onset of CHIKV infection or patients with a specific immune-mediated inflammatory pathology at follow-up [11,12]. Further, the pCHIK-CR patients were classified as cases of rheumatoid arthritis [36], spondylarthritis [37], systemic lupus erythematosus [38], post-viral arthritis [39], post-viral arthralgia [39], and soft tissue rheumatism (tenosynovitis, bursitis, fasciitis, noninflammatory localized pain, enthesitis, or fibromyalgia) [40]. The participants without rheumatic manifestations (Wo-RMs) at follow-up were classified as controls; this means the absence of persistent articular and extra-articular symptoms or the development of a specific immune-mediated inflammatory pathology during follow-up.

### 2.4. Immunological Factor Assays

We quantified the immunological factors in the samples collected during the baseline visit, which were stored at −80 °C. The serum concentrations of IL-4, IL-6, IL-8/CXCL-8, IL-27, CCL-2, CXCL-9, and CXCL-10 were quantified using the Milliplex^®^ Human Cytokine/Chemokine/Growth Factor Panel A (HCYTA-60K, Millipore, Billerica, MA, USA) kit on the Luminex MAGPIX^®^ system (XMAP Technology, MAGPIX, Austin, TX, USA), following the manufacturer’s instructions. Specific IgG antibodies against CHIKV were quantified using the NovaLisa^®^ IgG capture ELISA kit (NOVATEC Inmunodiagnostica GmbH, Dietzenbach, Germany). This assay measures NovaTec Units (NTUs), and the results are classified as negative (<9 NTU), positive (>11 NTU), or indeterminate (9–11 NTU).

### 2.5. Ethical Considerations

The Ethics Committees of the Universidad Industrial de Santander (Acta No. 07, 23 April 2021, and Acta No. 04, 18 March 2023) and the Centro de Atención y Diagnóstico de Enfermedades Infecciosas—C.D.I. (Acta 293, 9 July 2022) approved the study protocol. Informed consent was obtained from all the participants before inclusion in the original cohorts, as well as their authorization for the future use of the biological samples.

### 2.6. Data Analysis

We described continuous variables using the mean and standard deviation (SD), or the median and interquartile range [IQR] for those not normally distributed, according to the Shapiro–Wilk test. We calculated their absolute and relative frequencies (percentages) for discrete variables. We compared the means and medians between groups using Student’s *t*-test and the Kruskal–Wallis test, respectively. We assessed differences in proportions using the chi-square test and, alternatively, the Fisher’s exact test whenever the expected counts in the contingency tables were less than five. We assessed partial correlations among immunological factors concentrations using Spearman’s rank correlation coefficient (rs), adjusting for age at baseline and sex. We examined the shape of the functional relationship between each immunological factor and the case-control status using locally weighted regression (bandwidth = 0.75). Thresholds were defined as those points around which ≥3 first differences of consecutive, smoothed, predicted probabilities were consistently negative (indicating a decreasing trend) and positive (indicating an increasing trend) or vice versa. Then, we performed multiple logistic regression both with and without piecewise modeling (using the previously determined threshold) and estimated odds ratios (ORs) with 95% confidence intervals (95%CI), forcing the adjustment for age but conditioning it by age, sex, and disease duration at the baseline visit, but only if these covariates were associated to the case-control status at a significance level of ≤10% in the bivariate analysis. We evaluated the model fit using the Hosmer–Lemeshow (HL) test and their assessed discriminatory accuracy by estimating the area under the receiver operating characteristic (ROC) curve. Furthermore, we compared models with and without piecewise regression using the Akaike information criterion (AIC). The data analysis was conducted using the statistical software Stata/MP version 12.0 (Stata Corp., College Station, TX, USA).

## 3. Results

### 3.1. Population Characteristics

**Figure 1:**
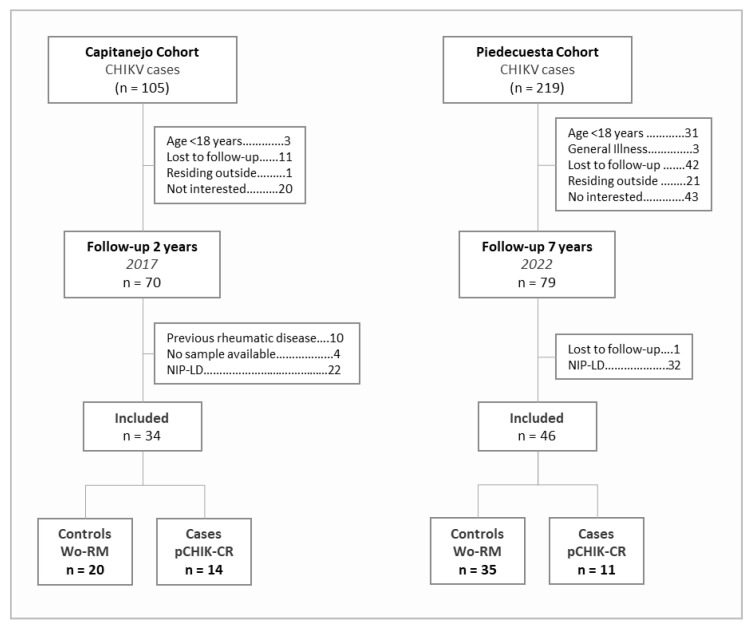
Flowchart of the case-control selection.

The analysis included 11 cases of pCHIK-CR and 35 controls from the Piedecuesta cohort and 14 cases and 20 controls from the Capitanejo cohort (Figure 1). Patients from both cohorts differed in the disease duration at which the immune factors were measured, the length of the follow-up, and their demographic and clinical characteristics (Table 1). The samples used for the immune factor quantification were collected earlier in the illness course in the Piedecuesta cohort compared to the Capitanejo cohort (2.8 versus 40.5 days, respectively); however, the follow-up for pCHIK-CR determination was longer in the Piedecuesta cohort (7.7 versus 2.2 years, respectively). Additionally, the Capitanejo cohort had a higher median age (54.0 versus 33.5 years), a greater proportion of women (73.5% versus 50.0%), and a higher prevalence of cardiovascular disease (20.6% versus 0.0%) and diabetes (7.1% versus 2.2%) than the Piedecuesta cohort. Furthermore, the cases were older and more likely to be women than the controls; however, these differences were statistically significant only in the Piedecuesta cohort. On the other hand, we screened the participants of the Piedecuesta cohort for coinfections with dengue and Zika viruses, but neither of them were detected.

### 3.2. Quantification and Correlation of Immunological Factors

**Figure 2:**
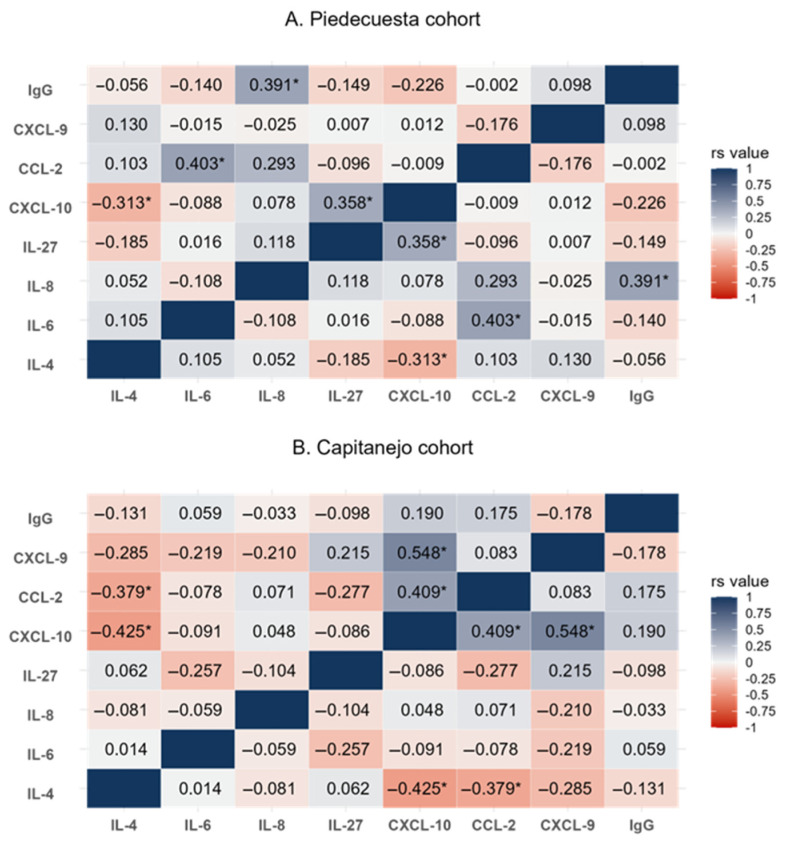
A heatmap of the partial correlations (adjusted for age and sex) between the immunological factors quantified at the baseline visit. rs value: = Spearman’s rank correlation coefficient; *p* < 0.05 (asterisk).

The levels of IL-4, IL-6, IL-27, CCL-2, CXCL-9, and CXCL-10 were higher in the Piedecuesta than in the Capitanejo cohort (Table 2 and Appendix A.1). In contrast, IL-8/CXCL-8 had higher concentrations in the Capitanejo cohort compared to the Piedecuesta cohort. On the other hand, the pattern of partial correlations between immunological factors differed across the cohorts (Figure 2). In the Piedecuesta cohort, CXCL-10 was positively correlated with IL-27 (rs = 0.358, *p* = 0.017) but negatively correlated with IL-4 (rs = −0.313, *p* = 0.039). Additionally, we observed positive correlations between IL-6 and CCL-2 (rs = 0.403, *p* = 0.007) and between IL-8/CXCL-8 and IgG (rs = 0.391, *p* = 0.009, Figure 2A). In the Capitanejo cohort, we observed statistically significant negative correlations between IL-4 and CXCL-10 (rs = −0.425, *p* = 0.019) and CCL-2 (rs = −0.379, *p* = 0.039) and positive correlations between CXCL-10 and CCL-2 (rs = 0.409, *p* = 0.025) and CXCL-9 (rs = 0.548, *p* = 0.002, Figure 2B).

### 3.3. Quantification of Immunological Factors by Case-Control Status

**Figure 3:**
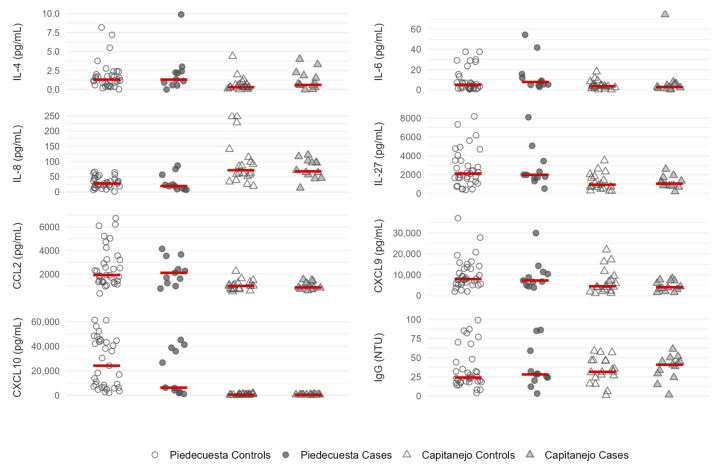
A scatter plot of immunological factor concentrations quantified at the baseline visit, stratified by the case-control status and the cohort. The Capitanejo cohort included 14 cases (pCHIK-CR) and 20 controls (Wo-RMs), while the Piedecuesta cohort included 11 cases of pCHIK-CR and 35 controls Wo-RMs. Horizontal red lines represent median concentrations.

In both cohorts, the baseline concentrations of IL-8/CXCL-8, CXCL-9, and CXCL-10 were non-statistically lower in the cases than in the controls, while the opposite was observed for IgG (Table 2 and Figure 3). In addition, the IL-6 and CCL-2 concentrations were higher in the samples collected during the acute phase of cases than in the controls (Piedecuesta cohort), whereas the IL-4 and IL-27 concentrations were higher in the samples collected during the subacute phase of the cases than in the controls (Capitanejo cohort); however, none of these differences reached statistical significance.

In the multivariate analysis, we found non-linear associations between IL-8/CXCL-8 and CXCL-10 concentrations—measured in the samples collected during the acute phase of CHIKV infection (Piedecuesta cohort)—and the likelihood of developing pCHIK-CR (Table 3 and Appendix A.2). The threshold concentrations were determined as 35.7 pg/mL for IL-8/CXCL-8 and 36,800 pg/mL for CXCL-10. For IL-8/CXCL-8, concentrations below the threshold indicated that a 1.0 pg/mL increase was associated with a 15% lower likelihood of pCHIK-CR (adjusted OR = 0.85; 95%CI: 0.74–0.99). On the other hand, for CXCL-10, a 100 pg/mL increase above the threshold was associated with a 6% lower likelihood of pCHIK-CR (adjusted OR = 0.94; 95%CI: 0.90–0.99). For both biomarkers, models with piecewise regression demonstrated a superior performance as compared to models without it. Additionally, we observed a non-linear association between CXCL-9 concentration—measured in samples collected during the subacute phase of CHIKV infection (Capitanejo cohort)—and the likelihood of pCHIK-CR; however, the model without piecewise regression had a similar performance with less complexity: a 100 pg/mL increase in CXCL-9 was associated with a 5% lower likelihood of pCHIK-CR (adjusted OR = 0.95; 95%CI: 0.91–0.99). No statistically significant associations, whether linear or non-linear, were observed for any other immunological factors.

## 4. Discussion

In this study, we observed that the concentrations of IL-8/CXCL-8, CXCL-9, and CXCL-10 were lower in the cases of pCHIK-CR compared to the controls; however, these differences were not statistically significant, regardless of whether the samples were collected during the acute or subacute phases. In contrast, the concentrations of IL-6 and CCL-2 were higher in samples collected during the acute phase of the cases of pCHIK-CR compared to the controls; however, these differences were not statistically significant. Additionally, IL-4 and IL-27 concentrations were elevated in the cases compared to the controls, during the subacute phase. A multivariate analysis indicated that in samples from the acute phase of CHIKV infection, levels of CXCL-10 (≥36,800 pg/mL) and IL-8/CXCL-8 (≤35.7 pg/mL) were associated with a lower likelihood of developing pCHIK-CR seven years after the symptoms’ onset. Further, CXCL-9 (≥6000 pg/mL) levels were associated with a reduced risk of pCHIK-CR after two years of follow-up in samples from the subacute phase.

We evaluated the hypothesis that immune factors are associated with the development of pCHIK-CR by analyzing data from two cohorts assembled during the CHIKV epidemic in Santander, Colombia, between 2014 and 2015. These cohorts differed in several aspects, including the duration of the disease when immune factors were measured, the length of the follow-up, and their baseline demographic and clinical characteristics. These differences help explain the varying incidence of pCHIK-CR between the cohorts [21,22,23,41], as indicated by their case-control ratios. This variation allowed us to explore the role of the immune response to CHIKV infection during the early stages of the disease, namely the acute and subacute phases.

During the acute phase of CHIKV, IL-8/CXCL-8 and CXCL-10 seem to play a significant role in predicting the development of pCHIK-CR. An increase of 1.0 pg/mL in IL-8/CXCL-8 levels (below the threshold of 35.7 pg/mL) was associated with a reduced likelihood of developing the outcome. However, no statistically significant association was observed at concentrations above this threshold. In terms of CXCL-10, higher levels (above 36,800 pg/mL) during the acute phase were associated with a lower probability of developing pCHIK-CR after seven years of follow-up. Similarly, in the subacute phase, higher levels of CXCL-9 above 6000 pg/mL were negatively associated with the likelihood of developing pCHIK-CR two years after the symptoms’ onset. CXCL-9 and CXCL-10 are STAT1-dependent inflammatory factors induced by different cell populations in response to interferons and IL-27 [42], and their expression correlates with the induction of interferon-stimulated genes (ISGs) which promote an antiviral state to control viral infections [24,42]. Additionally, both chemokines play a role in the recruitment of T cells and monocytes to infection sites, contributing to virus clearance and the inflammatory response [26,42,43]. Therefore, our findings suggest that the induction of early and strong IFN-STAT1-dependent inflammatory factors may enhance CHIKV control and prevent persistent joint symptoms.

In response to viral infections, the host induces an antiviral response characterized by the production of type I interferons (IFN-α, IFN-β), type III interferons (IFN-λ), and IL-27 (IFN-V) [42,44]. These interferons activate the JAK-STAT signaling pathway, triggering the transcription of ISGs that encode antiviral proteins (AVPs), cytokines, and chemokines essential for viral control and clearance [26,45]. Furthermore, type II IFN (IFN-γ) (produced by NK cells, CD4+ T cells (Th1), and cytotoxic CD8+ T cells) or IL-27 (from macrophages) induces the expression of IL-7, IL-15, CXCL-9, CXCL-10, and AVPs, contributing to the establishment of the antiviral state and controlling CHIKV replication [26,42]. Type I IFNs play a crucial role in controlling acute CHIKV infection, as mice lacking the type I IFN receptor exhibit higher viremia and increased susceptibility to severe CHIKV disease [46,47,48]. Additionally, CHIKV infection in human monocytes promotes a robust IFN-I-dependent antiviral response involved in controlling viral replication [49]. Considering this, if an efficient immune response for viral clearance occurs during the early days of CHIKV infection, the likelihood of progressing to the chronic phase of the disease could decrease, as well as the persistence of residual viral antigens in the synovial tissue and the associated inflammation.

In this study, the findings for CXCL-9 and CXCL-10, along with the absence of an IL-27 signal during the early stage of the disease, suggest that this response may be primarily mediated by the interferon signaling pathway, rather than IL-27, although IL-27 may activate the JAK-STAT signaling pathway independently of interferons and contribute to CCL-2, CXCL-9, and CXCL-10 production [24,26,42]. We did not find significant differences in IL-27 levels between the cases and the controls in our cohorts. Some studies have indicated that IL-27 serum levels positively correlate with the number of painful joints during the chronic phase of CHIKF (8.5 and 29 weeks), suggesting a potential role in later inflammatory stages [50].

No statistically significant association was found between early CCL-2 and IL-6 levels and the development of pCHIK-CR. This suggests that these factors are not differentially regulated in acute CHIKV infection based on the case-control status. Their release may be stimulated by signaling pathways other than JAK-STAT, such as the NF-kB pathway [51,52]. Moreover, their contribution to the disease’s pathogenesis may become evident during the chronic phase, potentially through the perpetuation of inflammation and tissue damage [51,53,54,55]. Previous studies assessing the relationship between IL-6 levels during the acute phase and joint pain at 12 and 20 months of follow-up found no significant association [27,56]. CCL-2 has not yet been evaluated using the methodology applied in our study.

We also found no statistically significant association for IL-4, despite its known role in modulating adaptive immunity. This finding contrasts with the results from Chan et al., who reported that a strong immune response during the acute phase, including TNF-α, IL-4, IL-2, and IL-13, reduced the likelihood of persistent joint pain after 20 months [56]. This discrepancy may be partially explained, on the one hand, by insufficient statistical power in our study and, on the other, by the use of a different definition of the outcome (joint pain versus pCHIK-CR). Additionally, IL-4 plays a key role in adaptive immunity, primarily by promoting B-cell proliferation and differentiation, which are essential for antibody production. Since humoral responses develop later in the disease’s natural history, IL-4 levels during the acute phase may not exhibit a distinct pattern based on the case-control status.

This study has some strengths worth mentioning. First, it is a nested case-control study within two cohorts that, given their differences in design (the duration of the disease when immune factors were measured and the length of follow-up), allowed us to explore the role of the immune response to CHIKV infection during the early stages of the disease, namely the acute and subacute phases. Further, the Piedecuesta cohort is among the studies conducted in Latin America with the most extended follow-up to assess pCHIK-CR, and additional follow-up visits could be conducted in this population. Second, the nested case-control approach minimized the risk of a selection bias, considering that the controls were selected from the same population as the cases. Third, outcome adjudication was conducted by certified rheumatologists who were unaware of the results from the immunological factor quantification, which, on the one hand, ensures the validity of pCHIK-CR determination and, on the other, minimizes the risk of an information bias. Fourth, we adjusted the associations under study for biologically and clinically relevant confounders, which minimized the risk of spurious findings. This study also has limitations. First, we only measured immunological factors at the baseline visit of each cohort, which precluded the evaluation of their dynamic interplay throughout disease progression to the chronic phase of the disease. Second, due to differences in the cohorts’ designs (including the timing of immune factor measurement, the follow-up duration, and the baseline demographic characteristics), we analyzed each cohort separately rather than combining the datasets. While this approach minimized a potential bias arising from cohort heterogenicity, it also limited the statistical power to detect associations between some immunological factors and pCHIK-CR due to the reduced sample size within each cohort. Third, using serological testing for CHIKV diagnosis in a single sample may carry a risk of false positives; however, the original cohorts were conducted during the early introduction of CHIKV in these municipalities, when prior immunity was unlikely. Finally, the CHIKV cases were identified in two municipalities in Colombia, which could limit the generalizability of the findings to other populations and epidemiological contexts.

Considering the limitations of this study and the still-incomplete understanding of the immunopathogenesis of chronic CHIKV-related disease, future research could benefit from additional follow-up visits to evaluate potential changes in the frequency of pCHIK-CR over time. Furthermore, the collection of serum, peripheral blood cells, and synovial membrane biopsies during the chronic phase could yield valuable insights into the expression of the immunological factors evaluated. Longitudinal studies with repeated measurements of these immune factors would also provide a better understanding of disease’s pathogenesis.

## 5. Conclusions

Our results suggest that an increase in the concentrations of IL-8/CXCL-8 and CXCL-10 during the acute phase and of CXCL-9 during the subacute phase of CHIKV infection may be associated with a reduced likelihood of developing pCHIK-CR. This observation suggests the possibility that early and strong immune responses could contribute to improved CHIKV control and potentially reduce the risk of persistent joint symptoms. Furthermore, these chemokines could be considered as candidate biomarkers for stratifying the risk of developing chronic sequels of CHIKV. These could enable timely symptom management and improve the quality of life of the patients. However, these results should be interpreted with caution and considered exploratory. Validation in more extensive studies is necessary to confirm their clinical relevance and to assess their potential utility in guiding early intervention strategies to improve patient outcomes.

## Figures and Tables

**Table 1:** Demographic characteristics of the CHIKV cases at the baseline visit, by their case-control status.

Characteristic, *n* (%)	Cases	Controls	Total	*p*
Piedecuesta cohort				
Female	9 (81.2)	14 (40.0)	23 (50.0)	0.035
Age (years)	45.0 [15.0]	30.0 [20.0]	33.5 [19.0]	0.001
Medical history				
Diabetes mellitus	0 (0.0)	1 (2.9)	1 (2.2)	1.000
Cardiovascular disease	0 (0.0)	0 (0.0)	0 (0.0)	-
Articular disease	0 (0.0)	0 (0.0)	0 (0.0)	-
No medical history	11 (100.0)	34 (97.1)	45 (97.8)	1.000
Capitanejo cohort				
Female	12 (85.7)	13 (65.0)	25 (73.5)	0.250
Age (years)	60.2 [13.3]	48.8 [27.3]	54.0 [25.5]	0.150
Medical history				
Diabetes mellitus	0 (0.0)	1 (10.0)	1 (7.1)	1.000
Cardiovascular disease	1 (25.0)	3 (27.3)	4 (20.6)	1.000
Articular disease	0 (0.0)	0 (0.0)	0 (0.0)	-
No medical history	13 (92.9)	17 (85.0)	30 (88.2)	1.000

The Capitanejo cohort included 14 cases (post-CHIKV chronic rheumatism, pCHIK-CR) and 20 controls (without rheumatic manifestations, Wo-RMs), while the Piedecuesta cohort included 11 cases of pCHIK-CR and 35 controls Wo-RMs. The figures in each cell represent medians [first and third quartiles] and absolute (relative) frequencies.

**Table 2:** Immunological factors quantified during the baseline visit by case-control status.

Biomarker (pg/mL)	Cases	Controls	Total	*p*	Median Difference	
					Absolute	Relative (%)
Piedecuesta cohort						
IL-4	1.3 [0.6–2.4]	1.3 [0.6–2.0]	1.3 [0.6 −2.2]	0.661	0.0	0.0
IL-6	7.7 [4.8–15.5]	4.9 [1.5–15.2]	5.4 [1.9–15.2]	0.102	2.8	57.1
IL-8/CXCL-8	19.1 [8.8–56.4]	27.3 [18.4–42.8]	23.4 [16.4–42.8]	0.421	−8.1	−29.9
IL-27	1978.0 [1631.9–3435.9]	2104.3 [1052.3–3513.3]	1981.8 [1307.3–3481.2]	0.867	−126.3	−6.0
CCL-2	2119.3 [1246.8–3552.7]	1929.9 [1339.7–3290.0]	2024 [1339.7–3290.0]	0.598	189.4	9.8
CXCL-9	7230.7 [4974.4–11,349.4]	7926.7 [5518.5–13,596.0]	7836.9 [5518.5–12,908.3]	0.709	−696.0	−8.8
CXCL-10	6333.5 [2862.6–38,829.6]	24,223.1 [6484.3–44,777.8]	21,293.3 [6329.2–43,769.0]	0.082	−17,889.6	−73.9
IgG *	28.0 [20.0–59.0]	24.0 [18.0–44.0]	25.0 [18.0–44.0]	0.699	4.0	16.7
Capitanejo cohort						
IL-4	0.6 [0.3–1.8]	0.3 [0.2–0.9]	0.6 [0.2–1.0]	0.268	0.3	116.7
IL-6	2.6 [1.2–5.9]	3.4 [1.8–5.6]	3.2 [1.4–5.9]	0.674	−0.8	−23.5
IL-8/CXCL-8	67.4 [45.2–96.9]	71.2 [53.4–105.8]	67.4 [50.9–97.9]	0.806	−3.8	−5.3
IL-27	1036.4 [814.7–1379.1]	927.7 [579.2–1907.2]	951.7 [680.6–1715.5]	0.834	108.7	11.7
CCL-2	878.9 [771.2–1259.6]	1002.8 [733.9–1187.1]	991.0 [737.7–1248.1]	0.944	−124.0	−12.4
CXCL-9	4032.6 [3046.9–7170.6]	4479.4 [2518.5–10,558.0]	4226.0 [2813.5–7558.3]	0.441	−446.8	−10.0
CXCL-10	522.9 [390.8–631.6]	589.0 [373.0–858.4]	536.4 [388.2–733.3]	0.382	−66.1	−11.2
IgG *	40.7 [29.1–44.7]	31.4 [23.7–46.1]	34.9 [25.2–46.0]	0.632	9.3	29.6

The Capitanejo cohort included 14 cases (post-CHIKV chronic rheumatism, pCHIK-CR) and 20 controls (without rheumatic manifestations, Wo-RMs), while the Piedecuesta cohort included 11 cases of pCHIK-CR and 35 controls Wo-RMs. The figures in each cell represent medians [first and third quartiles]. * Expressed in NovaTec Units (NTUs).

**Table 3:** Association between pro-inflammatory factors and post-CHIKV chronic rheumatism.

Biomarker	Crude OR (CI 95%)		Adjusted OR (CI 95%)	
	With Piecewise	Without Piecewise	With Piecewise	Without Piecewise
IL-8/CXCL-8 (pg/mL) *	-	1.00 (0.97–1.03)	-	0.98 (0.94–1.03)
<35.7	0.90 (0.81–0.98)	-	0.85 (0.74–0.99)	-
≥35.7	1.09 (1.01–1.18)	-	1.09 (0.97–1.22)	-
Age	-	-	1.13 (1.01–1.27)	1.15 (1.03–1.28)
Sex	-	-	0.13 (0.14–1.07)	0.16 (0.23–1.13)
Disease onset	-	-	0.47 (0.21–1.05)	0.61 (0.32–1.18)
HL	0.634	0.585	0.875	0.825
AUC	0.75 (0.59–0.91)	0.42 (0.19–0.65)	0.92 (0.85–1.00)	0.92 (0.85–1.00)
AIC	48.8	54.1	38.8	42.0
CXCL-10 (100 pg/mL) *	-	1.00 (0.99–1.01)	-	1.00 (0.99–1.01)
<36,800	1.00 (0.99–1.01)		1.01 (0.99–1.02)	-
≥36,800	0.98 (0.96–1.01)	-	0.94 (0.90–0.99)	-
Age	-	-	1.18 (1.03–1.35)	1.12 (1.02–1.22)
Sex	-	-	0.13 (0.02–1.14)	0.26 (0.04–1.62)
HL	0.502	0.226	0.830	0.969
AUC	0.57 (0.38–0.75)	0.68 (0.48–0.87)	0.90 (0.80–0.99)	0.87 (0.74–0.99)
AIC	53.3	53.2	39.5	42.8
CXCL-9 (100 pg/mL) †	-	0.99 (0.97–1.01)	-	0.96 (0.93–0.99)
<6000	1.03 (0.98–1.08)	-	0.98 (0.92–1.05)	-
≥6000	0.96 (0.90–1.02)	-	0.95 (0.91–0.99)	-
Age	-	-	1.10 (1.01–1.21)	1.11 (1.02–1.22)
Sex	-	-	0.29 (0.02–3.51)	0.22 (0.02–2.47)
Disease onset	-	-	0.96 (0.91–1.02)	0.97 (0.91–1.02)
HL	0.682	0.133	0.578	0.598
AUC	0.64 (0.45–0.83)	0.58 (0.38–0.78)	0.84 (0.70–0.98)	0.85 (0.71–0.98)
AIC	46.4	47.3	41.9	40.5

* Piedecuesta cohort: 11 cases of pCHIK-CR and 35 controls Wo-RMs. † Capitanejo cohort: 14 cases of pCHIK-CR and 20 controls Wo-RMs. HL: *p*-value corresponding to the Hosmer–Lemeshow goodness-of-fit test. AUC: area under the curve. AIC: Akaike information criterion.

## Data Availability

The data supporting the findings of this study are available upon reasonable request. Due to privacy and ethical restrictions, the data cannot be made publicly available.

## Appendix A

### Appendix A.2. The Exploration of the Functional Relationship Between IL-8/CXCL-8, CXCL-9, and CXCL-10 and the Case-Control Status

The Capitanejo cohort includes 14 cases (chronic post-CHIKV rheumatism, pCHIK-CR) and 20 controls (without rheumatic manifestations, Wo-RMs), whereas the Piedecuesta cohort includes 11 cases of pCHIK-CR and 35 controls Wo-RMs. The exploration of IL-8/CXCL-8 and CXCL-10 was conducted in the Piedecuesta cohort, while the exploration of CXCL-9 was performed in the Capitanejo cohort.
